# Tissue accumulation of neutrophil extracellular traps mediates muscle hyperalgesia in a mouse model

**DOI:** 10.1038/s41598-022-07916-8

**Published:** 2022-03-09

**Authors:** Kazuaki Suzuki, Masahiro Tsuchiya, Shinichirou Yoshida, Kazumi Ogawa, Weijian Chen, Makoto Kanzaki, Tadahisa Takahashi, Ryo Fujita, Yuqing Li, Yutaka Yabe, Toshimi Aizawa, Yoshihiro Hagiwara

**Affiliations:** 1 Department of Orthopaedic Surgery, Graduate School of Medicine, Tohoku University, Sendai, Japan; 2 Graduate School of Biomedical Engineering, Tohoku University, Sendai, Japan; 3 Department of Nursing, Tohoku Fukushi University, 6-149-1 Kunimi-ga-oka, Sendai, 981-3201 Japan

**Keywords:** Chronic inflammation, Molecular medicine

## Abstract

Accumulation of uric acid (UA) during muscular trauma is a factor involved in the development of muscle hyperalgesia. Neutrophil extracellular traps (NETs), DNA-based reticular structures to capture UA, play a central role in the pain onset of gout attacks; however, the involvement of NETs via the elevation of local UA level in muscle hyperalgesia due to injuries from muscle overuse remains unknown. The triceps surae muscles (TSMs) in the unilateral hindlimb of mice were electrically stimulated to induce excessive muscle contraction. Mechanical withdrawal thresholds, tissue UA levels, neutrophil recruitment, and protein amount of citrullinated histone 3 (citH3), a major marker of NETs, were investigated. Furthermore, whether neutrophil depletion, extracellular DNA cleavage, and administration of the urate-lowering agent febuxostat improved muscle hyperalgesia caused by NET formation was examined. CitH3 expression upon neutrophil recruitment was significantly increased in the stimulated TSMs with increased tissue UA levels, whereas febuxostat administration improved muscle hyperalgesia with decreased citH3 and tissue UA levels, as observed in neutrophil depletion and extracellular DNA digestion. The underlying mechanism of muscle hyperalgesia associated with locally recruited neutrophils forming NETs due to increased tissue UA levels potentially plays a significant role in creating a vicious circle of muscle pain.

## Introduction

Skeletal muscle is a common source of pain that markedly impairs activities of daily living^1^. It is widely observed as a major sign of various pathologies, such as neck and shoulder pain^2^, nonspecific lower back pain^3^, and myofascial pain syndrome (MPS)^4^. Based on the higher lifetime prevalence of skeletal muscle pain among the labor population^5,6^, muscle overuse is a key pathogenic event in developing muscle pain. Fundamentally, overuse trauma prompts muscle fibers to release extracellular adenosine triphosphate (ATP), which directly activates pain signaling through purinergic and metabotropic receptors via autocrine and paracrine functions^7–10^. Indeed, both the serum and muscle tissue levels of uric acid (UA), an end product of purine nucleotides including ATP and dead cell DNA, are reportedly increased due to the production of damaged muscle fibers^11–15^.

UA has recently been recognized as a damage-associated molecular pattern (DAMP), which activates an intracellular complex called the inflammasome for processing and releasing interleukin (IL)-1β and IL-18^16^. Our recent study using a muscle pain model by the repeated electrical stimulation of the triceps surae muscles (TSMs) revealed the marked recruitment of inflammatory cells, including neutrophils and macrophages, which produce proinflammatory cytokines, such as IL-1β and IL-18, due to inflammasome activation caused by the increase in tissue UA levels^11,15,17^. Furthermore, based on the improvement of muscle hyperalgesia by the administration of xanthine oxidase inhibitors, we suggested that a higher tissue UA concentration could be a causative factor of mechanical hyperalgesia^11^. Thus, the dysregulated innate immune response with hyperuricemia has been associated with gout among various autoinflammatory diseases, which are characterized by unprovoked episodes of recurrent or continuous inflammation in the absence of high-titer autoantibodies or antigen-specific lymphocytes^18–20^.

A recent study has also supported that the dysregulated innate immune response of neutrophils against monosodium urate (MSU) crystals in the gout flare is a part of the autoinflammatory response because of the involvement of neutrophil extracellular traps (NETs)^21,22^. The release of NETs, a unique defense mechanism continuing from cell death (NETosis), is regarded as a valuable target for disease pathogenesis in gout^21,23,24^. NETs are primarily composed of their own DNA released as reticular structures with an oxidative burst in order to capture and eliminate pathogens, including DAMPs^23,24^, and citrullinated histone H3 (citH3) plays a central role in NETosis^22^. Interestingly, NETs also contribute to aggregating neutrophilic proinflammatory mediators, thereby limiting, yet prolonging the inflammatory status^24^. Indeed, NETs are not only DAMPs after degradation, but also a potential source of UA with extracellular nucleotide metabolism^24^. Thus, research findings regarding the underlying mechanism of UA accumulation with a focus on NETs in damaged muscle tissues will facilitate the development of an integrative therapeutic strategy for chronic muscle pain.

Although the involvement of NET-mediated processes in numerous painful diseases, including gout and rheumatoid arthritis, has been reported^21–23^, no reports have described the relationship between NETs and muscle pain. Therefore, we aimed to examine whether neutrophils played a key role in developing muscle pain via NET production caused by increased UA levels, using a muscle pain model with muscle overuse.

## Results

### MSU stimulation induced NETs and muscle pain in the TSMs

**Figure 1:**
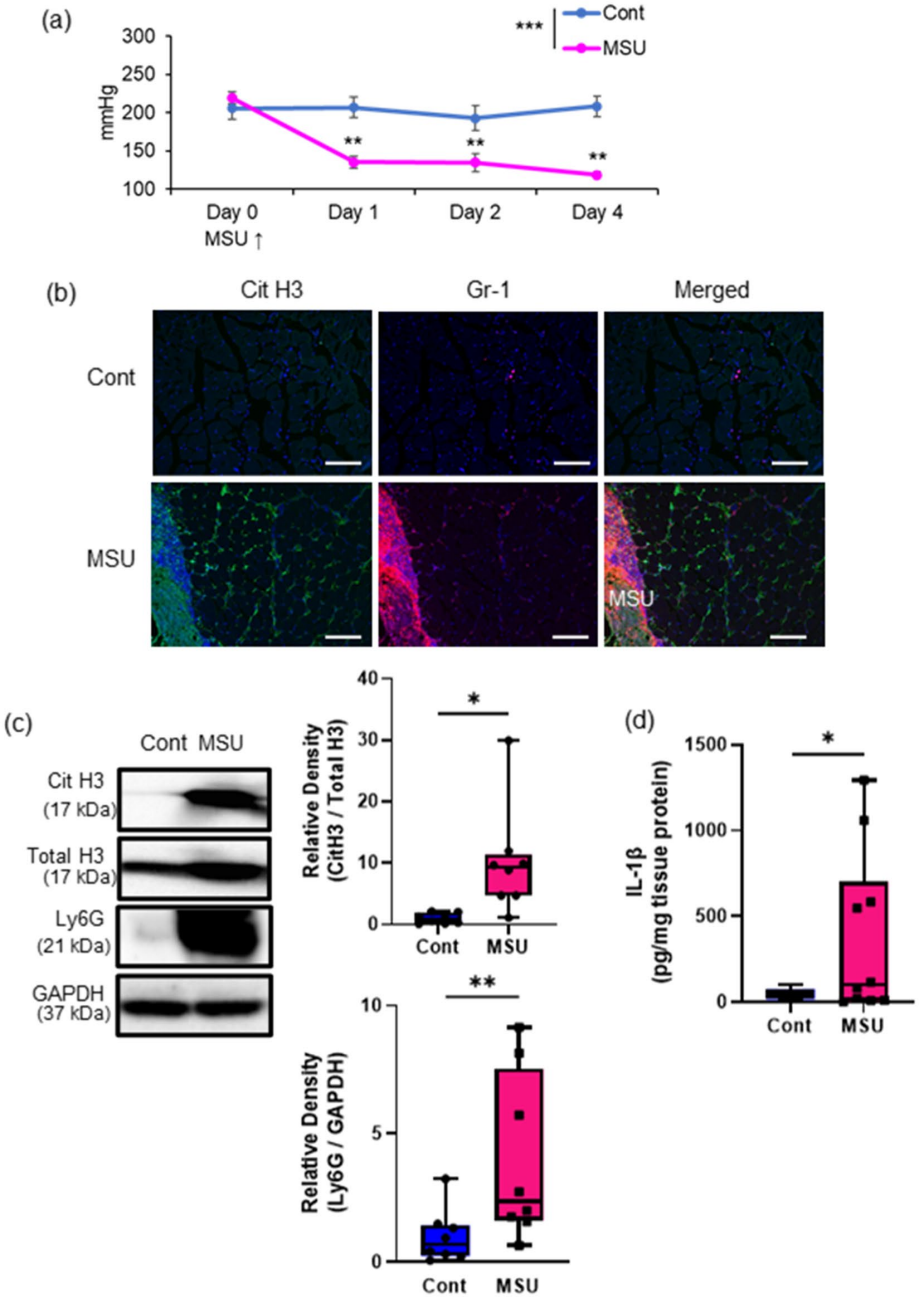
Induction of muscle hyperalgesia and NETs with intramuscular MSU injection. (**a**) Time-series experiment of the MNT values in the MSU- or saline-injected (contralateral) hindlimbs (n = 6 in each group). (**b**) Representative IHC images (200 ×) of TSM tissue on day 2, indicating citH3 (green), Gr-1 (red), and DAPI (blue). (**c**) Western blotting analysis of citH3 and Ly6G amounts in the TSM tissues on day 2 after intramuscular injection of MSU or saline (n = 8 in each group). The relative density is normalized based on the loading control (total histone H3: total H3 and GAPDH, respectively). Scale bar = 100 μm. (**d**) ELISA analysis was performed to evaluate protein levels of IL-1β, using MSU- or saline-injected TSM tissues on day 2 (n = 10 in each group). All data are shown as Tukey’s boxplots with individual data points and analyzed using paired t-test or two-way ANOVA with Tukey’s post-hoc multiple comparison test for time-series of MNT values. Statistical significance is indicated with * (p < 0.05), ** (p < 0.01), and *** (p < 0.001).

To investigate whether MSU crystals induced NETs in skeletal muscle tissues, as shown in previous studies, we first performed the intramuscular injection of MSU by following a method reported in our previous study (Fig. 1)^11^. Compared to those of the control group, the mechanical nociceptive threshold (MNT) of the MSU group significantly decreased after the intramuscular injection of MSU (Fig. 1a). In terms of IHC observations, control TSMs with saline injection did not show any changes, whereas increased immunoreactivity of citH3 and Gr-1 was located around the outer edge of MSU (Fig. 1b & Fig. S1a). Western blotting using the TSM tissues stimulated with MSU on day 2 showed significant increases in citH3 and Ly6G (Fig. 1c). Additionally, IL-1β expression in the TSMs injected with MSU significantly increased on day 2 (Fig. 1d & Fig. S1b).

### Excessive muscle contraction caused by repeated EPS induced mechanical hyperalgesia with elevated NET production in the skeletal muscle tissues

**Figure 2:**
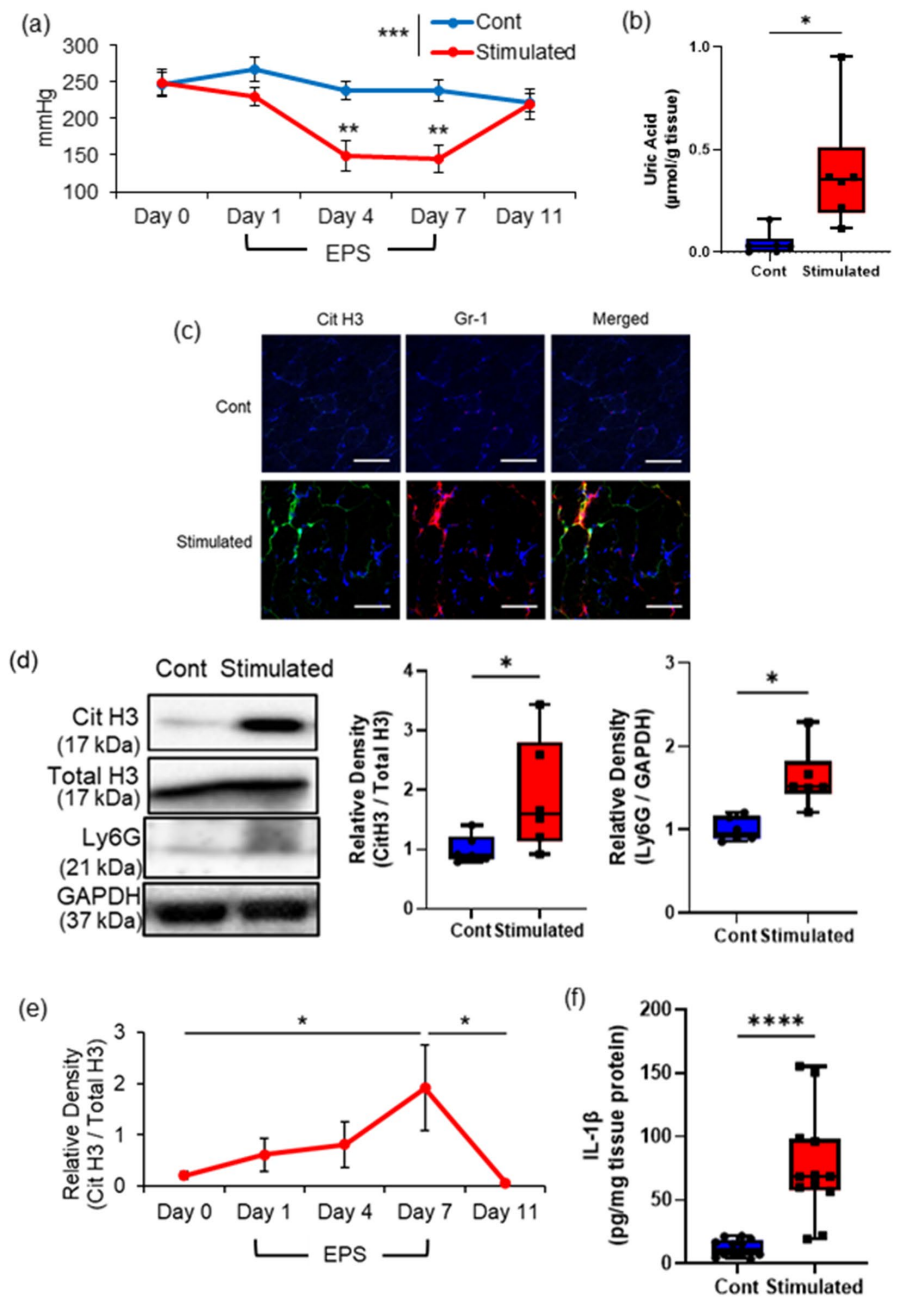
Effects of repeated electrical stimulation (EPS) to induce muscle hyperalgesia and NETs. (**a**) Time series experiment of the MNT values in the stimulated and non-stimulated hindlimbs (contralateral side in the same mice; n = 6 in each group). (**b**) The uric acid level in the stimulated and non-stimulated TSM tissues on day 7 (n = 6 in each group). (**c**) Representative IHC images (200 ×) of mouse TSM tissue with or without repeated EPS on day 7, indicating citH3 (green), Gr-1 (red), and DAPI (blue). Scale bar = 50 μm. (**d**) citH3 and Ly6G amounts in the TSM tissues with or without repeated EPS on day 7 by western blotting analysis (n = 6 in each group). The relative density is normalized based on the loading control (total histone H3: total H3 and GAPDH, respectively). (**e**) Time series experiment of citH3 induction in stimulated TSM tissues. After normalization of citH3 using the total H3, a ratio of citH3 in the ipsilateral side relative to the contralateral side was calculated in each individual (n = 4 in each group). (**f**) ELISA analysis was performed to evaluate protein levels of IL-1β, using the TSM tissues of control or stimulated mice on day 7 (n = 12 in each group). All data are shown as Tukey’s boxplots with individual data points and analyzed using paired t-test to compare the mean of two different samples or two-way ANOVA followed by Tukey’s post-hoc multiple comparison test for time-series experiment of MNT values and for comparing more than two groups. Statistical significance is indicated with * (p < 0.05), ** (p < 0.01) and *** (p < 0.001), **** (p < 0.0001).

We next confirmed whether excessive muscle contraction by EPSs induced NET formation associated with an increase in tissue UA levels (Fig. 2). As previously shown in our studies^11,17^, the MNTs of the stimulated TSMs significantly decreased due to repeated stimulation, compared to those of the contralateral muscle, and were significantly decreased, with the minimum MNT noted on day 7 (Fig. 2a). Reduced MNTs in the stimulated TSMs increased again after 7 days of electrical stimulation, and MNTs between the control (non-stimulated) and EPS-stimulated groups were not significant on day 11 (Fig. 2a). The tissue samples on day 7 were mainly used in our analyses based on the above results. Among the TSM samples on day 7, tissue UA levels in the stimulated TSMs were significantly higher than those in the contralateral TSMs (Fig. 2b).

IHC analysis indicated distinct distribution patterns of citH3 immunoreactivities, which colocalized with Ly6G immunoreactivities in the stimulated TSMs, but not in the non-stimulated TSMs (Fig. 2c & Fig. S2a). Hematoxylin and eosin staining showed typical skeletal muscle tissue histology in both groups (data not shown). Further confirming these observations, western blotting indicated that the relative density of citH3 and Ly6G (Fig. 2d) normalized to the loading control (total H3 and GAPDH, respectively) were significantly increased in the stimulated TSMs on day 7 compared with those in the non-stimulated TSMs. The time-series experiment of citH3 protein in comparison with ipsilateral to contralateral TSM tissues during and after EPS is shown in Fig. 2e. The protein amount ratio of citH3 showed the highest value on day 7 with repeated EPS, whereas it recovered to the level on day 0 after stopping the stimulation. IL-1β mRNA expression was significantly increased in the stimulated TSMs than in the non-stimulated TSMs (Fig. 2f & Fig. S2b). Notably, the plasma level of citH3 was not significant between control and EPS-stimulated mice (Fig. S2c).

### DNase treatment ameliorated muscle hyperalgesia and NET induction owing to repeated EPS

**Figure 3:**
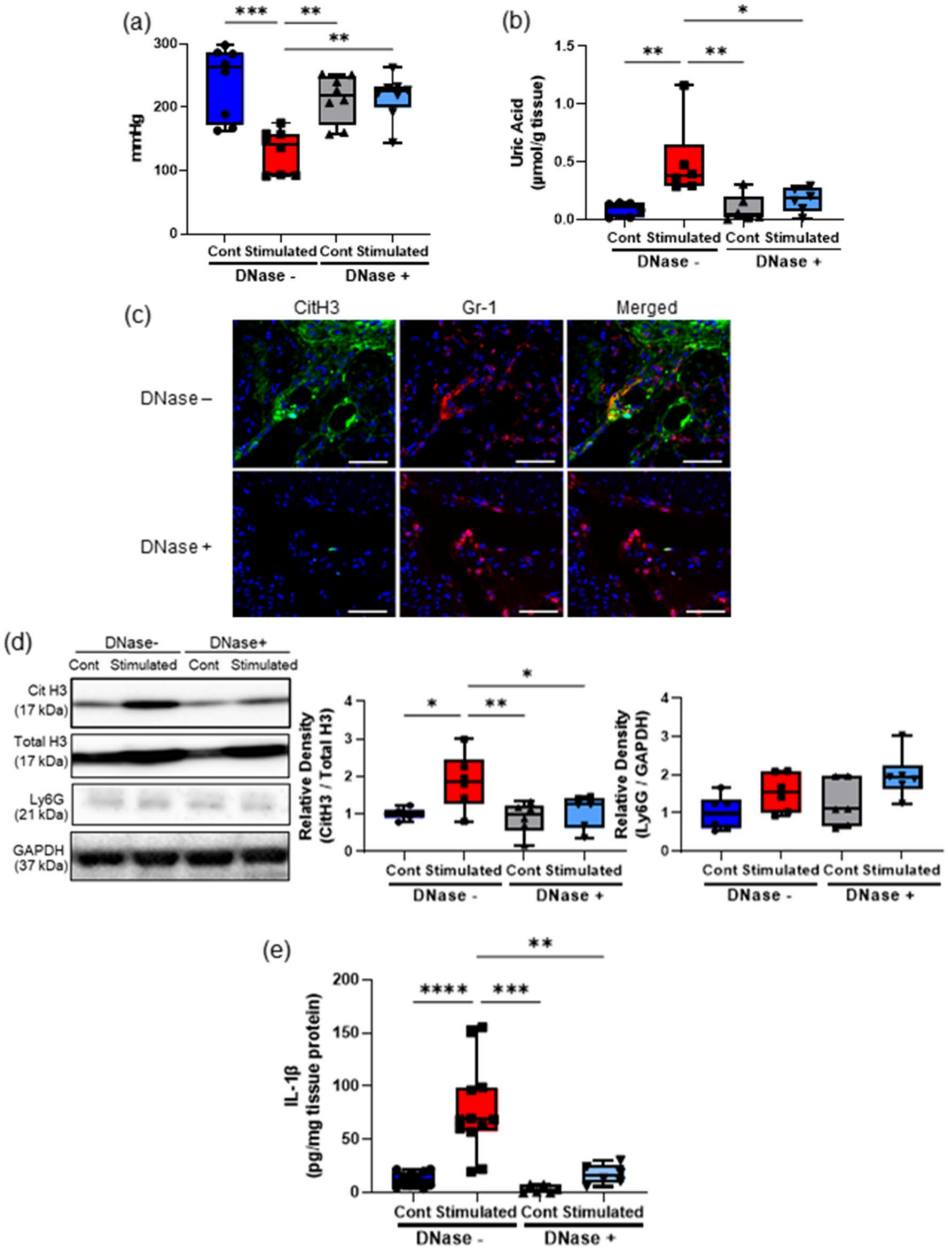
Effects of DNase treatment on NETs induction associated with muscle hyperalgesia. The MNT values of hindlimbs (**a** n = 8 in each group) and the uric acid level (**b** n = 6 in each group) in the TSM tissues in sham-stimulated control and stimulated mice injected intravenously with 10 mg/kg/day of DNase I or a vehicle. (**c**) Representative IHC images (200 ×) of the stimulated TSM tissue with or without DNase I administration on day 7, indicating citH3 (green), Gr-1 (red), and DAPI (blue). Scale bar = 50 μm. Western blotting analysis of citH3 (**d**) and Ly6G amounts in TSM tissues on day 7 (n = 6 in each group). The relative density is normalized based on the loading control (total H3 and GAPDH). (**e**) ELISA analysis was performed to evaluate protein levels of IL-1β in TSM tissues on day 7 (n = 12 mice without DNase treatment, n = 6 mice with DNase treatment). All data are shown as Tukey’s boxplots with individual data points and analyzed using unpaired t-test for comparing the mean of two different samples or two-way ANOVA followed by post-hoc Tukey’s test for a comparison of more than two groups. Statistical significance is indicated with * (p < 0.05), ** (p < 0.01) and *** (p < 0.001), **** (p < 0.0001).

To confirm the pathological significance of NETs in muscle hyperalgesia, we administered DNase I to degrade extracellular DNA, including NETs, and demonstrated its impact on muscle hyperalgesia (Fig. 3). Intravenous administration of DNase I significantly increased the MNT values (Fig. 3a) and decreased tissue UA levels (Fig. 3b) in the stimulated TSMs. Consistent with these observations, IHC (Fig. 3c) and western blotting analyses (Fig. 3d and e) indicated that DNase I administration decreased citH3 expression, but not the protein amount of Ly6G. Additionally, IL-1β mRNA expression in the stimulated TSMs decreased following DNase I administration (Fig. S3b). Moreover, DNase administration reduced not only citH3, but also tissue UA levels despite the same intensity and duration of the repeated EPS application.

### Neutrophil depletion ameliorated muscle hyperalgesia and NET induction owing to repeated EPS

**Figure 4:**
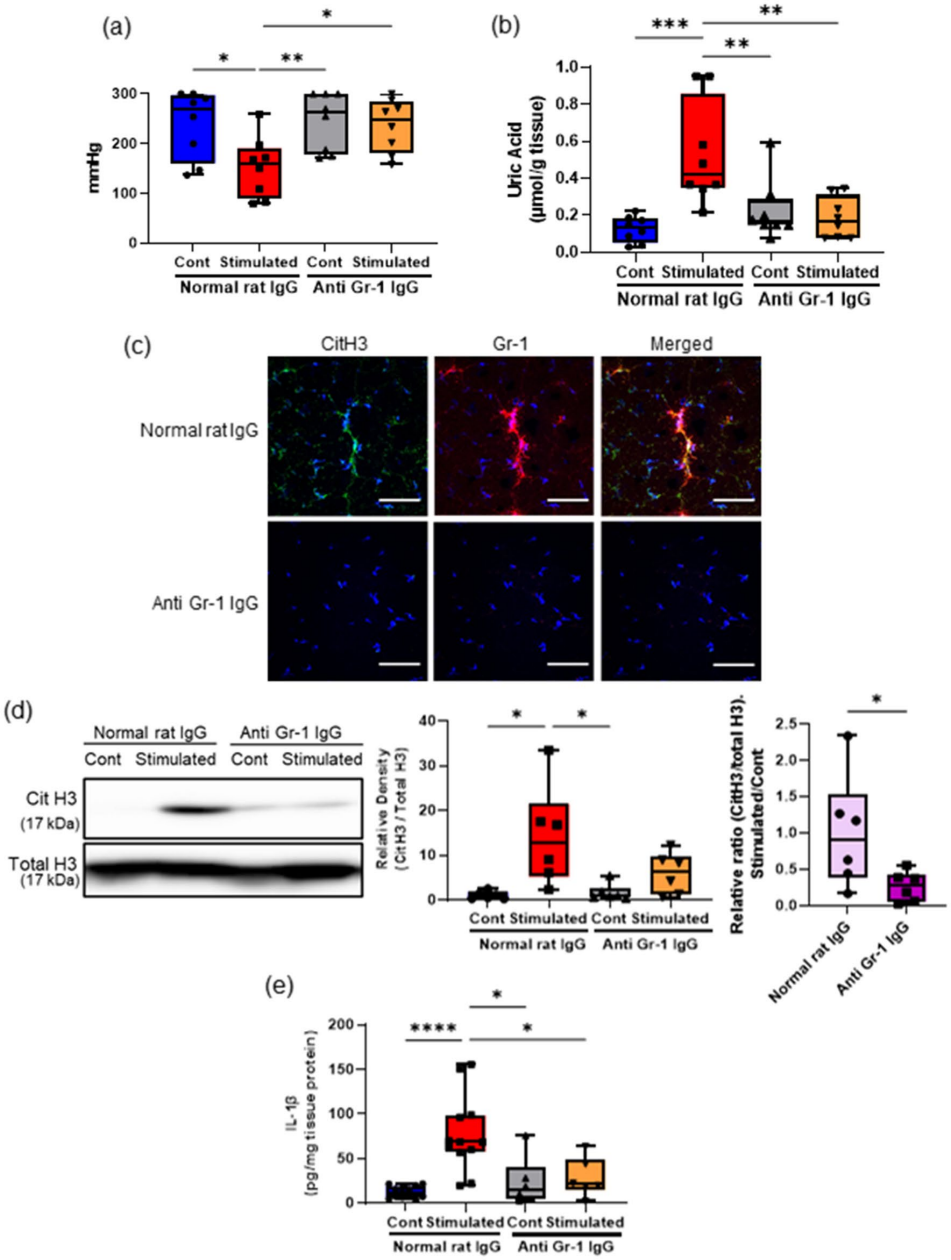
Effects of neutrophil depletion on NETs induction associated with muscle hyperalgesia. The MNT values of hindlimbs (**a**) and the uric acid level (**b**) in the TSM tissues in sham-stimulated control and stimulated mice injected intravenously with 5 mg/kg BW of anti-Gr-1 antibodies or control rat IgGs every 3 days (N = 8). (**c**) Representative IHC images (200 ×) of the stimulated TSM tissue with or without neutrophil depletion on day 7, indicating citH3 (green), Gr-1 (red), and DAPI (blue). Scale bar = 50 μm. (**d**) Western blotting analysis of citH3 amounts in TSM tissues on day 7 (N = 6). The relative density is normalized based on the loading control (total H3), and further calculated as a ratio in the ipsilateral side relative to the contralateral (unstimulated) side of the same individual. (**e**) ELISA analysis was performed to evaluate the protein levels of IL-1β in the TSM tissues on day 7 (n = 12 mice with control IgG administration, n = 6 mice with neutrophil depletion). All data are shown as Tukey’s boxplots with individual data points and analyzed using unpaired t-test for comparing the mean of two different samples or two-way ANOVA followed by post-hoc Tukey’s test for a comparison of more than two groups. Statistical significance is indicated with * (p < 0.05), ** (p < 0.01) and *** (p < 0.001).

Given the absolute necessity of neutrophil recruitment for NET induction, we next examined the effects of an experimental neutrophil depletion by prior treatment with anti-Gr-1 antibody on muscle hyperalgesia (Fig. 4). Neutrophil depletion resulted in a significant increase of the MNT in the stimulated hindlimbs (Fig. 4a) and reduced tissue UA levels (Fig. 4b) in the TSMs with repeated EPS. Similar to the IHC images showing weaker immunoreactivity of citH3 and Gr-1 in the neutrophil-depleted group than in the control group (Fig. 4c), neutrophil depletion significantly decreased the relative ratio of citH3 (Fig. 4d) and IL-1β mRNA expressions (Fig. 4e) in the stimulated TSMs compared with the control mice injected with the same amount of normal rat IgG. Notably, the tissue UA level in the stimulated TSMs was decreased with experimental neutrophil depletion.

### Febuxostat treatment relieved muscle hyperalgesia due to repeated EPS

**Figure 5:**
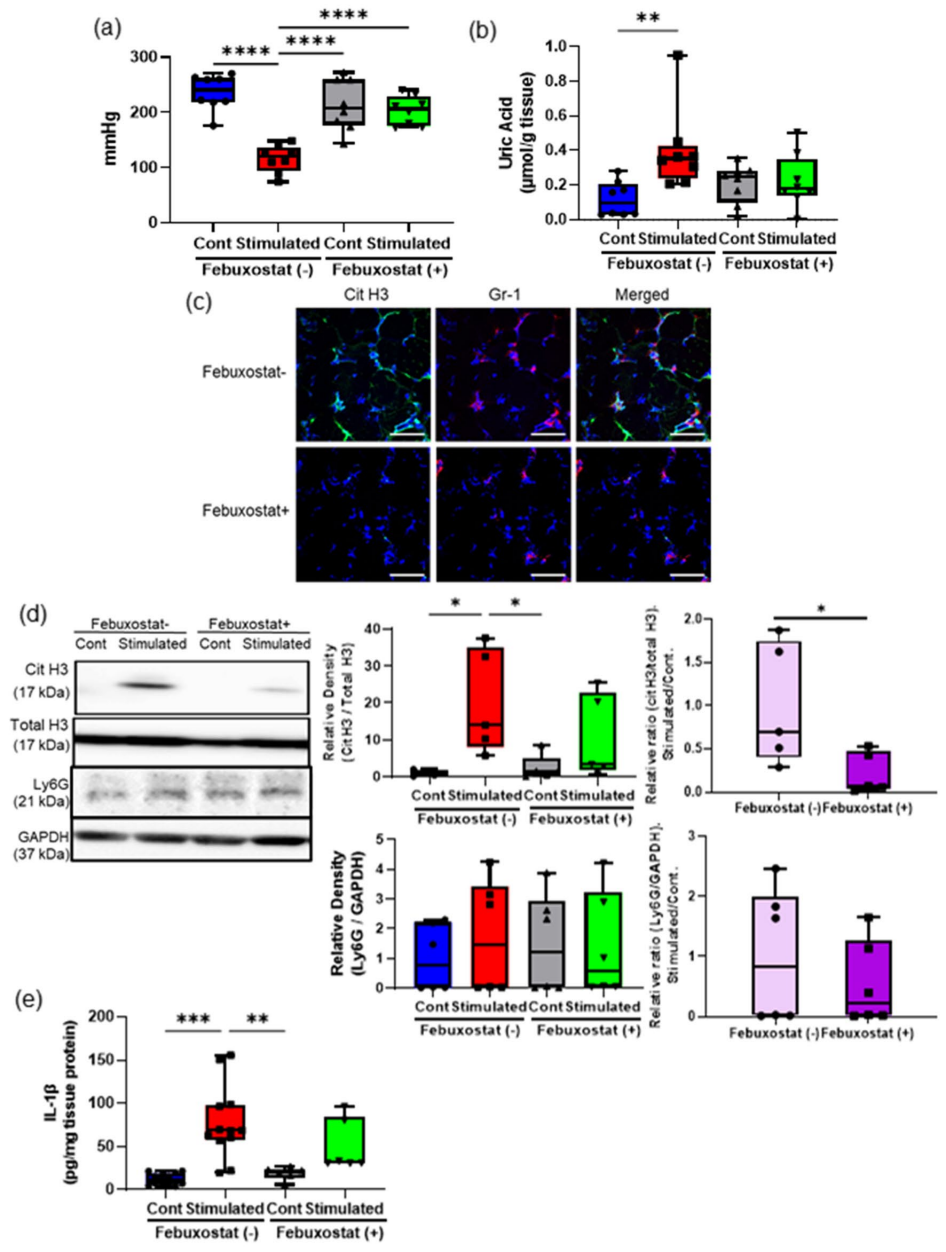
Effects of febuxostat treatment on NETs induction associated with muscle hyperalgesia. The MNT values of hindlimbs (**a**) and the uric acid level (**b**) in the TSM tissues in sham-stimulated control and stimulated mice injected intraperitoneally with 5 mg/kg BW/day of febuxostat or a vehicle (N = 8). (**c**) Representative IHC images (200 ×) of the stimulated TSM tissue with or without febuxostat treatment on day 7, indicating citH3 (green), Gr-1 (red), and DAPI (blue). Scale bar = 50 μm. (**d**) Western blotting analysis of citH3 amounts in the TSM tissues with or without febuxostat treatment on day 7 (N = 6). The relative density is normalized based on the loading control (total H3), and further calculated as a ratio in the ipsilateral side relative to the contralateral (unstimulated) side of the same individual. (**e**) ELISA analysis was performed to evaluate the protein levels of IL-1β in the TSM tissues on day 7 (n = 12 mice with a vehicle injection, n = 6 mice with febuxostat treatment). All data are shown as Tukey’s boxplots with individual data points and analyzed using unpaired t-test to compare the mean of two different samples or two-way ANOVA followed by post-hoc Tukey’s test for a comparison of more than two groups. Statistical significance is indicated with * (p < 0.05), ** (p < 0.01), and *** (p < 0.001).

Since our recent study reported that the decrease in tissue UA levels resulted in the improvement of muscle hyperalgesia^11^, we investigated whether the administration of febuxostat, a xanthine oxidase inhibitor, influenced NET induction due to repeated EPS (Fig. 5). As reported in our previous study^11^, febuxostat treatment increased the MNTs (Fig. 5a), and reduced tissue UA levels (Fig. 5b) and IL-1β mRNA expressions (Fig. S5b) in the stimulated TSMs. Consistent with these observations, IHC (Fig. 5c) and western blotting analyses indicated that febuxostat administration reduced citH3 expression (Fig. 5d) but not neutrophil recruitment (Fig. 5c and e) in the stimulated TSMs. These results indicate that high tissue UA levels play a central role in NET induction in skeletal muscle tissues with hyperalgesia.

### MPM imaging with in vivo staining for NET-like structures

**Figure 6:**
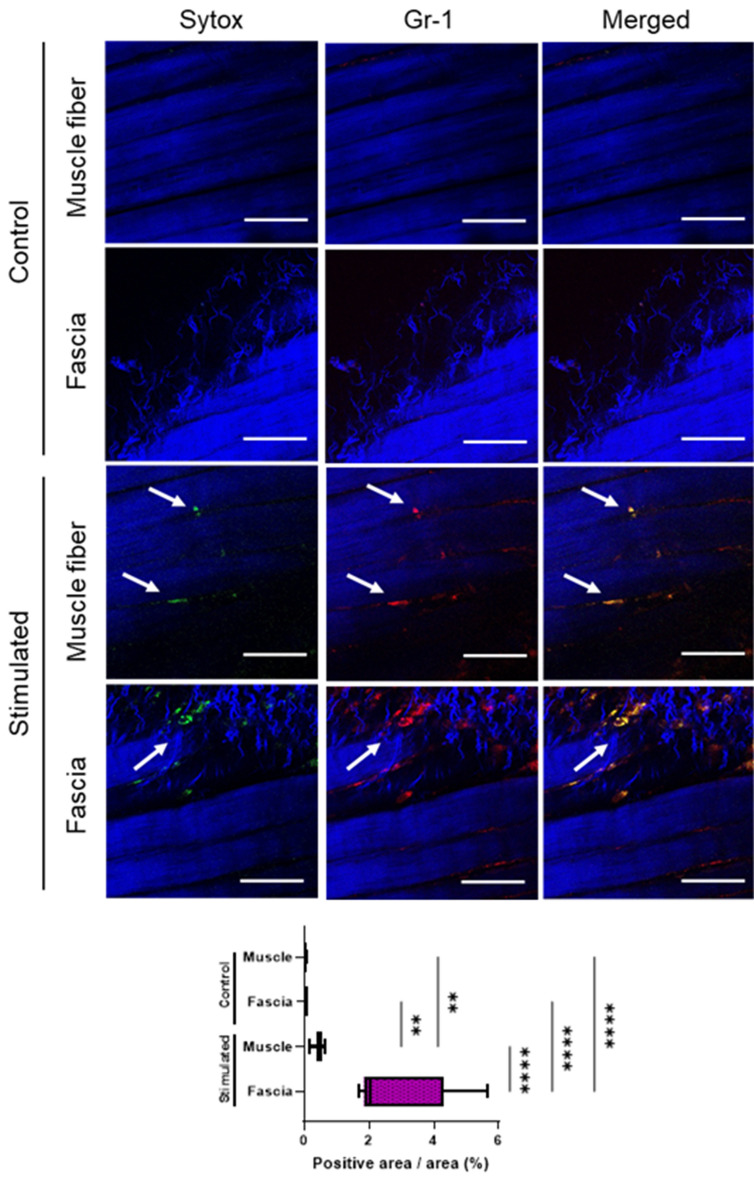
MPM imaging of neutrophil recruitment releasing extracellular DNA due to repeated electrical stimulation (EPS) as assessed by in vivo immunostaining. Representative images (200 ×) show fluorescence colocalization of neutrophils (indicated by white arrows) labelled with QD655-conjugated anti-Gr-1 antibody (red) and extracellular DNA labelled with SYTOX (green) in the TSM tissues (muscle fibers and myofascial zone) of sham-control or stimulated mice on day 7. Muscle and collagenous fibers are visualized with second-harmonic generation (blue). The graph summarizing area quantification (%) of fluorescence colocalizations within myofascial or muscle fibers of the TSM tissues is indicated. Data are shown as Tukey’s boxplots with individual data points (n = 3). The quantification results were analyzed using one-way ANOVA with Tukey’s post hoc test. Scale bar = 50 μm. Statistical significance is indicated with ** (p < 0.01) and **** (p < 0.0001).

To confirm that repeated EPS induced neutrophil recruitment with extracellular DNA release in TSM tissues, MPM imaging was performed using an in vivo immunostaining method^25^. Repeated EPS significantly increased the fluorescence of neutrophils and extracellular DNA in TSM tissues, as visualized by QD655 and SYTOX, respectively (Fig. 6 and Supplemental movie). In particular, the myofascial region showed significantly more fluorescence colocalizations than the TSM myofibers.

## Discussion

**Figure 7:**
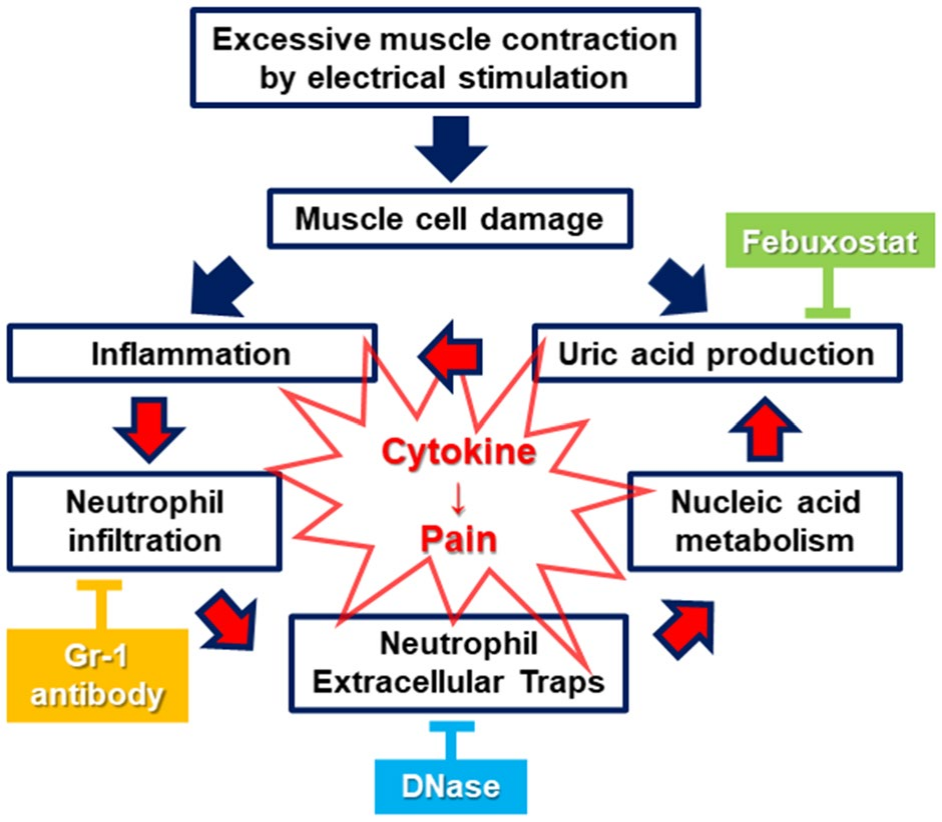
A simple schema of the developing pathway of muscle hyperalgesia caused by a higher tissue uric acid level in the mouse model, and the effects of inhibitors at each factor. Muscle hyperalgesia associated with locally recruited neutrophils forming NETs due to the increased tissue UA levels might create a “vicious circle” because of prolonging unfavorable inflammatory environments with new neutrophil infiltration.

The present study clarified the potential pathogenesis of NETs in muscle hyperalgesia (exhibiting elicited nocifensive behaviors) due to a locally higher tissue UA level caused by excessive muscle contraction (Fig. 2) as well as noted the observations in the TSMs with MSU injection (Fig. 1). Our findings showed a potentially “vicious cycle” model of muscle hyperalgesia due to a higher tissue UA level, which reciprocates between neutrophil recruitment and NET induction (Fig. 7).

NETs play an essential function in immobilizing and killing pathogens, including bacteria, while limiting inflammation, whereas inappropriate NET release has late harmful effects on tissues due to the release of cytotoxic and proinflammatory mediators with NET degradation^22–24^. Delayed-onset muscle soreness, a typical muscle pain experienced after excessive exercise, persists for a few days^7^. Based on the increased serum NET levels after acute severe exercise^26^, NETs not only play a crucial role in prolonging and strengthening local inflammation and pain, as observed in our results, but also in general muscle physiology. Although plasma citH3 levels were not increased with repeated EPS-stimulation in our study, future studies using other biomarkers, such as MPO-DNA complex, are warranted to clarify the systemic effects of NETs associated with hyperalgesia. Additionally, recent studies have focused on the implications of NETs in numerous autoinflammatory diseases, including systemic lupus erythematosus, psoriasis, and gout^21,23,27^. Interestingly, our results also demonstrated that febuxostat administration, as well as the experimental neutrophil depletion and DNase administration, improved the local NET accumulation associated with muscle hyperalgesia (Fig. 3). Febuxostat reduces not only tissue UA content but also neutrophil recruitment in damaged tissues^28^. Thus, local NET accumulation in skeletal muscles damaged by repeated excessive exercise would enhance inflammation and pain with a sustainable supply of proinflammatory stimuli consisting of inflammatory mediators, such as IL-1β and DAMPs, including UA.

MSU crystallization due to hyperuricemia is a typical causative stimulus of acute and painful inflammatory responses in gout attacks^20,21^. Since our previous study showed that a higher UA level in skeletal muscle tissues was a potential trigger for muscle hyperalgesia^11^, we indicated in this study that NETs are crucial mediators in developing muscle hyperalgesia caused by a higher UA content in skeletal muscle tissues. However, no MSU crystals were found in the skeletal muscle tissues with excessive contraction. In terms of MSU crystallization, Martillo et al. indicated that 405 μmol/L was the solubility limit of the serum UA^29^. Our results showed that the average local UA concentration (approximately 500 μmol/L) in the stimulated muscles was higher than the abovementioned value. Because Braga et al. indicated that high concentrations of soluble UA directly activated innate immunity^29^, a higher tissue UA level possibly contributes to the modulation of a microenvironment with a higher risk of NET induction as neutrophil recruitment. One key limitation of this study is that mice have urate oxidase, a uricase mostly expressed in the liver that degrades uric acid, which contributes to their resistance to hyperuricemia^29,30^. Due to the role of endogenous uricase in enhancing local accumulation of uric acid in mouse muscle tissue, the hyperalgesia caused by local hyperuricemia observed in the present study might be stronger in human muscle tissue. Thus, future studies examining this issue in human muscle tissue are warranted.

In terms of a vicious cycle of a higher tissue UA level leading to NET accumulation and consequent development of muscle hyperalgesia (Fig. 7), neutrophil depletion (Fig. 4), DNase treatment (Fig. 3), and febuxostat treatment (Fig. 5) essentially resulted not only in the improvement of muscle hyperalgesia but also in the reduction of the tissue UA levels in the damaged skeletal muscles. Interestingly, DNase administration improved NETs accumulation and tissue UA levels. Since DNase prevents vascular occlusion by NETs^31^, it might be associated with the maintenance of the microcirculation in damaged muscle tissues. Based on the activation of the inflammasome pathway in macrophages by a higher UA level^11,16^, the neutrophil–macrophage interactions as the main innate immune response definitively contribute to the deterioration of muscle hyperalgesia. Although future studies with appropriate designs are warranted to clarify the interactions between neutrophils and macrophages, these findings suggest that extracellular DNA consisting of NETs with neutrophil recruitment is potentially the main source inducing higher UA levels in skeletal muscle tissues via purine metabolism and plays a role in exacerbating muscle hyperalgesia.

Furthermore, MPM imaging of NET-like structures showed that extracellular DNA fragments were mostly colocalized with neutrophils but not in the skeletal muscle fibers. Shinoda et al. also reported no histological changes in the stimulated skeletal muscle tissues under the same experimental conditions^10^. Thus, a higher tissue UA content with skeletal muscle overloading may predominantly depend on neutrophil recruitment followed by NETosis. Furthermore, neutrophils releasing extracellular DNA were predominantly recruited more in myofascial tissues than in muscle fibers (Fig. 6). This is consistent with the multiple lines of evidence regarding the pathomechanisms underlying the development of MPS with the characteristic irritable response in myofascial tissues^15^. Future studies are warranted to clarify the mechanisms that dominantly recruit neutrophils in myofascial tissues to provide new insights regarding MPS therapy and management.

As a key limitation in the study, mice have urate oxidase, namely uricase, which is mostly expressed in the liver that degrades uric acid and contributes to the resistance to hyperuricemia^29,30^. However, due to the role of endogenous uricase in mice muscle tissues for improving the local accumulation of uric acid, the hyperalgesia caused by local hyperuricemia examined in the present study might be stronger, especially when developing in human muscle tissue. Thus, future studies examining human muscle tissues are warranted.

## Conclusions

Our findings clarified the underlying mechanism of muscle hyperalgesia associated with recruited neutrophils forming NETs, which potentially cause higher UA levels in skeletal muscle tissues. Thus, the regulation of local UA metabolism while focusing on NET induction would be a potential therapeutic target to relieve muscle pain.

## Methods

### Experimental animals

Forty-nine male BALB/c mice (5–7 weeks old) were used in this study (CLEA Japan, Tokyo, Japan). The study was carried out in compliance with the ARRIVE guidelines. Further, the experimental design, care, and use of the mice were performed according to the guidelines for animal experiments at Tohoku University. Ethical approval for this study was obtained from the Animal Research Committee of Tohoku University (approval number: 2019 MdA-070). The mice were kept in standard cages maintained in an air-conditioned room at 23 °C ± 1 °C with a 12-h light–dark cycle with ad libitum access to standard food pellets and tap water, in accordance with the National Standards Relating to the Care and Management of Laboratory Animals and Relief of Pain (Notification No. 88 of the Ministry of the Environment, Japan, April 28, 2006). General anesthesia was induced in each mouse by the intraperitoneal injection of medetomidine (0.3 mg/kg; ZENOAQ, Fukushima, Japan), midazolam (4.0 mg/kg; SANDZ, Tokyo, Japan), and butorphanol (5.0 mg/kg; Meiji Seika Pharma Co, Tokyo, Japan). Mice were sacrificed by cervical dislocation under inhalation anesthesia with isoflurane (MSD Animal Health, Kenilworth, NJ, USA).

### Repeated electrical stimulation of the triceps surae muscles

Electrical pulse stimulation (EPS) was repeatedly applied to induce excessive muscle contraction of the TSMs, as previously described^10,11,17^. Two stainless electrodes (single-stranded stainless-steel wire, A-M system, Sequim, WA, USA) were transcutaneously inserted into the proximal and distal ends of the TSMs on the dorsal surface of the hindlimbs under anesthesia. EPS using a STG4004 multichannel system (MCS GmbH, Reutlingen, Germany) was performed on the muscle at 10 Hz with a 10-V amplitude and a 100-µs pulse width for 30 min per day (day 0 to 6). Bilateral hindlimbs were immobilized with full ankle dorsiflexion using a scotch tape to stabilize the static muscle tension during EPS. Electrodes were also applied to the contralateral hindlimbs without EPS. Within 24 h after the last EPS, TSMs were collected, immediately frozen in liquid nitrogen, and stored at -80 °C until assayed.

### Assessment of mechanical nociceptive thresholds (MNTs)

MNT was defined as the amount of pressure required to evoke pain-related reactions such as vocalization, struggling, and hindlimb withdrawal. MNT was evaluated using the Randall–Selitto test (MK-201D Pressure Analgesy-Meter, Muromachi Kikai Co., Tokyo, Japan)^32^. The test was performed with a cone-shaped, 2.6-mm-diameter tip attached to a scale with a display. Pressure, gradually increasing at regular intervals (10 mmHg/s) with setting the cut-off value to be 300 mmHg, was applied to the lateral side of the TSMs^11^. Since circadian rhythm affects pain sensitivity, all MNT measurements were performed in the morning. To avoid bias, MNT assessments were analyzed by an investigator who was blinded to the experimental conditions.

### Local effect of monosodium urate stimulation

To confirm the local effects of MSU on NET induction, recrystallized MSU (No. 133–13,432; Wako Pure Chemicals Industries, Osaka, Japan) dissolved in saline was administered to the right TSMs (MSU group), as previously described^33,34^. Saline was administered to the contralateral TSMs (control group). The solution (100 μL) of recrystallized MSU (200 μg) was injected under the fascia of the lateral head of the TSMs using a 27-gauge needle. MNTs were assessed on days 0, 1, 2, and 4 post-injection. TSMs were obtained, frozen in liquid nitrogen, and stored at -80 °C until assayed.

### Pharmacological experiments related to neutrophil extracellular trap formation

We administered deoxyribonuclease (DNase) I (Wako Pure Chemical Industries, Ltd., Osaka, Japan; 10 mg per kg body weight [BW] per day) intravenously via the tail vein^35^ or febuxostat (F0847, Tokyo Chemical Industry Co., Ltd., Tokyo, Japan; 5 mg per kg BW per day) intraperitoneally^36,37^. The control mice were injected with the same volume of the control vehicle (saline) 15 min prior to the experiment.

To deplete neutrophils, anti-granulocyte-differentiation antigen 1 (Gr-1; a major neutrophil marker) antibody (RB6-8C5, rat IgG2b) purified from the culture supernatants of a hybridoma (provided by Dr. R. Coffman) was intravenously administered into the mice (Gr-1 group) once every 3 days at a dose of 5 mg/kg BW^37,38^ because the injection causes neutropenia for at least three days^39,40^. The control mice were also injected with an equivalent amount of normal rat IgG (Jackson Laboratories, Bar Harbor, ME) (IgG group).

### Measurement of tissue uric acid levels

The frozen muscles (30 mg) were homogenized in a 300 µL of lysis buffer (30 mM Tris, 100 mM sodium chloride, 1 mM ethylenediaminetetraacetic acid, 1% Triton X-100, 2.5 mM sodium fluoride, 2 mM sodium polyphosphate, 1 mM sodium orthovanadate, 1 mM phenylmethylsulphonyl fluoride, 10 μg/mL aprotinin, 1 μg/mL pepstatin, and 5 μg/mL leupeptin). The lysate was then centrifuged at 12,000 × g for 15 min at 4 °C. Supernatants were collected for the assay of tissue UA levels and western blotting. Tissue UA levels were measured using an assay kit (Cell Biolabs, Inc., San Diego, CA, USA), following the manufacturer’s instructions. All reagents, including uric acid standard solution, was freshly prepared before use. Each sample and standard were assayed in duplicate. After incubating for 20 min at 37 °C with the protection from light, fluorescence signals were measured at 595 nm for emission after excitation at 550 nm with a SpectraMax M5 microplate reader (Molecular Devices; MDS Analytical Technologies).

### Quantitative reverse transcription polymerase chain reaction

Total RNA was extracted from the TSM tissues using TRIzol (Molecular Research Centre Inc., Cincinnati, OH, USA). cDNA was prepared using a Transcriptor First Strand cDNA Synthesis Kit (Roche, Basel, Switzerland). The primer sequences used were as follows: IL-1b: F5’-TGG TGG GGG TTC TCT GTG GTT-3’ and R5’-TTG AGG CGG CTT TCT TTG TCC-3’ and EF1a1 (internal control primer): F5’-TCG CTT TGC TGT TCG TGA C-3’ and R5’-TGG GGT GGC AGG TGT TAG-3’. The relative expression levels of each mRNA were calculated as a function of the EF1a1 expression, as previously described^11,17^.

### Immunohistochemistry

TSM tissues were snap-frozen in liquid nitrogen and embedded in a Tissue-Tek OCT compound (Sakura Finetek, Tokyo, Japan). The cryosections were transversely cut into 5-µm thickness using a cryostat (CM1850; Leica, Nussloch, Germany) and mounted on coated glass slides, followed by acetone fixation. Then, the cryosections were washed and incubated with a blocking buffer (5% bovine serum albumin [BSA] in Tris-buffered saline with 0.1% Tween-20), including 10% normal goat serum (Nichirei Biosciences Inc., Tokyo, Japan) for 30 min. The slides were incubated with a polyclonal rabbit anti-citH3 antibody (ab5103; 10 ng/mL; Abcam, Waltham, MA, USA) and anti-Gr-1 antibody (RB6-8C5, rat IgG2b; 10 ng/mL; BioLegend, San Diego, CA, USA) in a blocking buffer for 2 h at room temperature (RT). Subsequently, the slides were incubated for 1 h with an Alexa Fluor 488-conjugated goat anti-rabbit IgG (A-11034, Life Technologies, Carlsbad, CA, USA; dilution, 1:750) for anti-citH3, and an Alexa Fluor 555-conjugated goat anti-rat IgG (A- 21,434, Life Technologies; dilution, 1:750) for Gr-1 at RT. After washing, the slides were incubated with 4,6-diamidino-2-phenylindole (Sigma–Aldrich; dilution, 1:500) for nuclear staining. Images were captured using a fluorescence microscope (Olympus FV1000; Olympus, Tokyo, Japan) equipped with an oil-immersion objective lens (UApo/340 40 × /NA 1.35). Images were analyzed using Fiji/ImageJ software (NIH, Bethesda, MD, USA). At least three images from each slide were captured at 200 × magnification. To avoid bias, a few animals were used for immunohistochemistry (IHC), and two slides/animals were analyzed. After confirming the reproducibility, representative images were obtained. We obtained the fluorescence localization of citH3 and Gr1 to quantitatively measure the accumulation of NETs in each area using ImageJ software^41,42^.

### Immunoblotting

The lysates extracted from the TSM tissues were adjusted to 4.0 mg/mL with a lysis buffer using a BCA protein assay kit (Thermo Fisher Scientific, Waltham, MA, USA). Then, 30 μL of samples were loaded for 5–12% SDS–polyacrylamide gel electrophoresis and transferred to Immobilon-P polyvinylidene difluoride membranes (Merck, Kenilworth, NJ, USA)^38^. The protein-transferred membranes were blocked with 5% BSA in Tris-buffered saline with 0.1% Tween-20 and incubated at 4 °C overnight with anti-citH3 (ab5103; 2 µg/mL), anti-histone H3 (ab1791; 1 µg/mL; Abcam), anti-Ly6G (127,602; 2 µg/mL; Biolegend), and anti- GAPDH (#2118; 1:1000 dilution; Cell Signaling, Beverly, MA, USA). The membranes were washed, followed by incubation at RT for 1 h with HRP-conjugated secondary antibodies (1:10,000 dilutions of #ab6734; Abcam; and 1:5,000 dilutions of #32,460; Thermo Fisher Scientific). Then, a signal using a chemiluminescence reagent, was obtained from SuperSignal West Femto Maximum Sensitivity Substrate (Thermo Fisher Scientific), and the band intensity was detected using the Image Quant TL system (GE Healthcare, Chalfont St Giles, UK). The relative density was normalized based on the loading control (total H3 and GAPDH) and was further calculated as a ratio in the ipsilateral side relative to the contralateral (unstimulated) side of the same individual to adjust a large individual difference.

### Enzyme-linked immunosorbent (ELISA) assays

The lysates extracted from the TSM tissues used for the immunoblotting were utilized for the measurement of IL-1b protein levels using Mouse IL-1 beta/IL-1F2 DuoSet ELISA (DY401; R&D) after being adjusted to 5.0 mg/mL using a BCA protein assay kit, according to the manufacturer's instructions. Furthermore, plasma levels of citrullinated histone H3 were determined using Citrullinated Histone H3 (Clone 11D3) ELISA kit (Cayman Chemicals). Blood samples were centrifuged at 3,000 rpm for 5 min at 4℃, and the supernatant was stored at -80℃ until use. The colorimetric signals were measured at 450 nm with a microplate reader.

### Multiphoton microscopy imaging using in vivo staining of NET-like structures

To detect NETs through in vivo staining, two types of fluorescent reagents, anti-Gr-1 antibody conjugated to Qdot655 (QD-Gr-1Ab, 3.0 μg per mouse) to detect neutrophils and SYTOX Green (Thermo Fisher Scientific, 1 µL of 5 mM solution per mouse) to detect extracellular DNA, were used^25,43,44^. Anti-Gr-1 antibodies were prepared by conjugation to Qdot 655 using the SAIVI rapid antibody labeling kit (Invitrogen). The conjugated antibodies were purified using a size-exclusion column, and its concentration was determined by measuring the absorbance at 679 nm. After 24 h from EPS on day 7, QD-Gr-1Ab and SYTOX were intravenously injected 20 min before animal sacrifice under anesthesia. Mice were fixed by transcardiac perfusion with 4% paraformaldehyde in phosphate buffered saline, and the TSM tissues were subjected to multiphoton microscopy (MPM) imaging. An upright A1R-MP multiphoton microscope (Nikon) equipped with a Ti–sapphire laser (Mai-Tai Deep See, Spectra-Physics), GaAsP non-descanned detectors, and a water-immersion objective lens (CFP75 Apo LWD 25x/NA1.1) were used for image recording with an excitation laser consistently set at 920 nm, with an area size of 510 μm × 510 μm and a resolution of 600 dpi. The wavelengths for detection using emission filter cubes were 492/SP nm for second-harmonic generation signals (blue channel), 525 ± 50 nm for SYTOX (green channel), and 629 ± 56 nm for QD-Gr-1Ab (far-red channel). By distinguishing between the muscle and myofascial area based on the second-harmonic generation signals indicating muscle and collagenous fibers^44^, we obtained the fluorescence colocalization of QD-Gr-1Ab and SYTOX to quantitatively measure the accumulation of NET-like structures in each area using the ImageJ software^42^. Furthermore, representative 3D image stacks focusing on neutrophil recruitment releasing extracellular DNA were reconstructed from the sequential images in the TSM structure (Movie S1 & S2).

### Statistical analysis

Statistical analyses were performed using SPSS (IBM, Armonk, NY, USA). Analysis of the MNT time-course data was performed using two-way analysis of variance (ANOVA), and repeated measurements were compared using Tukey’s post-hoc multiple-comparison test. To compare data from more than two groups from single and multiple days, one-way and two-way ANOVA with Tukey’s post-hoc multiple comparison tests were used, respectively. Western blotting and IL-1β data between the two groups were analyzed using the Wilcoxon signed-rank test. Other data between two groups were analyzed using paired t-tests. All data are expressed as the mean ± standard error. Statistical significance was set at p < 0.05.

### Ethics declarations

The experimental design, care, and use of the mice were performed according to the guidelines for animal experiments at Tohoku University. Ethical approval for this study was obtained from the Animal Research Committee of Tohoku University (approval number: 2019 MdA-070).

## Supplementary Information

Supplementary Figures.

Supplementary Video 1.

Supplementary Video 2.

Supplementary Video Legend.

Supplementary Information.

## Data Availability

The data that support the findings of this study are available from the corresponding author, MT, upon reasonable request.
