# Supplementary material for: Tissue accumulation of neutrophil extracellular traps mediates muscle hyperalgesia in a mouse model
Source: Sci Rep. 2022 Mar 9;12:4136. doi: 10.1038/s41598-022-07916-8 (PMC8907237; doi:10.1038/s41598-022-07916-8)
Supplement: Supplementary file 1 — Supplementary Figures. [file 41598_2022_7916_MOESM1_ESM.docx]

Figure S1


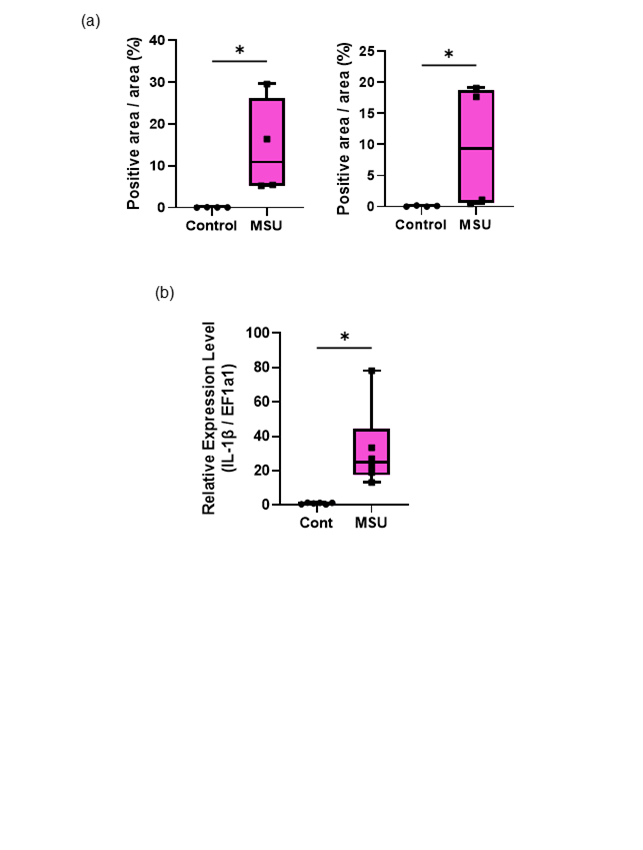


Figure S1. NETs induction with intramuscular MSU injection.

(a) Quantification of citH3 (left) and Gr1-positive areas (right) in the IHC images of the TSM tissues on day 2 after intramuscular injection of MSU or saline (n=4). (b) qRT-PCR analysis was performed to evaluate the expression levels of IL-1β using MSU- or saline-injected TSM tissues on day 2 (n=6). All data are shown as Tukey’s boxplots with individual data points. Statistical significance by paired t-test is indicated with * (p<0.05).

Figure S2


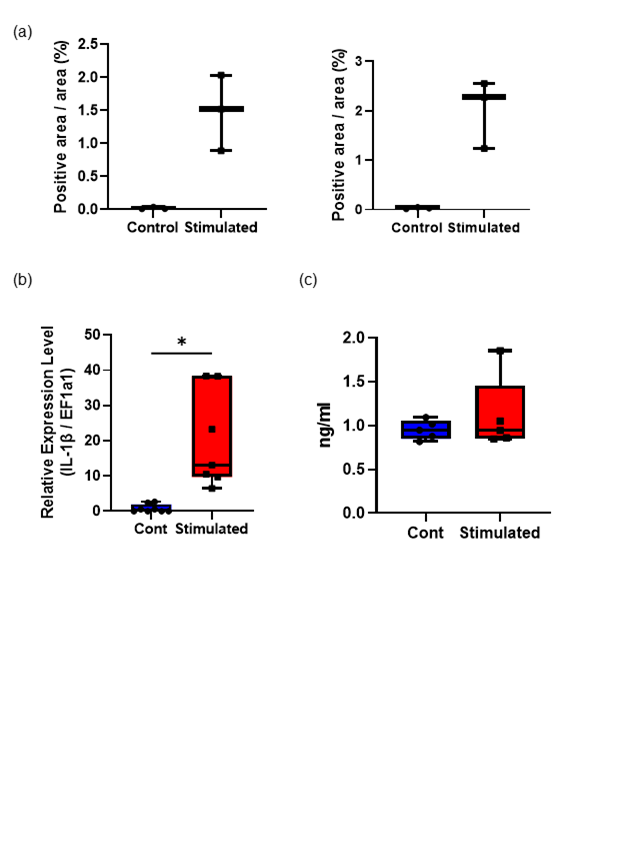


Figure S2. Effects of repeated electrical stimulation (EPS) to induce NETs and IL-1β expression in the TSM tissues associated with muscle hyperalgesia.

(a) Quantification of citH3 (left) and Gr1-positive areas (right) in the IHC images of the stimulated and non-stimulated TSM tissues on day 7 (n = 3). (b) qRT-PCR analysis was performed to evaluate expression levels of IL-1β in the TSM tissues with or without repeated EPS on day 7 (n=7). (c) The citH3 concentration of plasma determined by ELISA (n=5). All data are shown as Tukey’s boxplots with individual data points. Statistical significance by paired t-test is indicated with * (p<0.05).

Figure S3


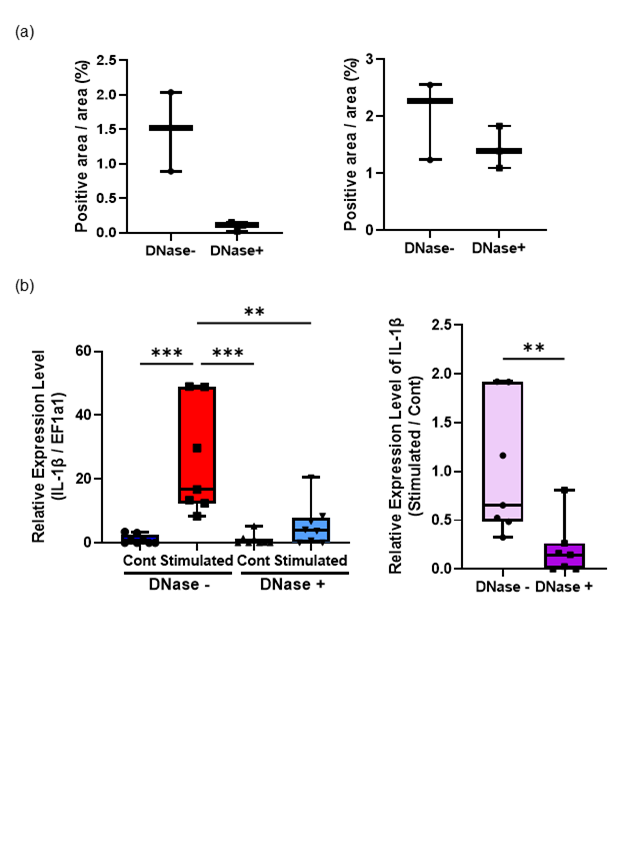


Figure S3. Effects of DNase treatment on NETs and IL1β expression in the TSM tissues associated with muscle hyperalgesia.

(a) Quantification of citH3 (left) and Gr-1 positive-areas (right) in the IHC images of the stimulated TSM tissues on day 7 with intravenous injection of 10 mg/kg/day of DNase Ⅰ or a vehicle (n = 3). (b) qRT-PCR analysis was performed to evaluate the expression levels of IL-1β in the TSM tissues on day 7 in sham-stimulated control and stimulated mice injected intravenously with 10 mg/kg/day of DNase Ⅰ or a vehicle (n=7), and was further calculated as a ratio in the ipsilateral side relative to the contralateral (unstimulated) side of the same individual. All data are shown as Tukey’s boxplots with individual data points. Statistical significance by paired t-test is indicated with ** (p<0.01) and *** (p<0.001).

Figure S4


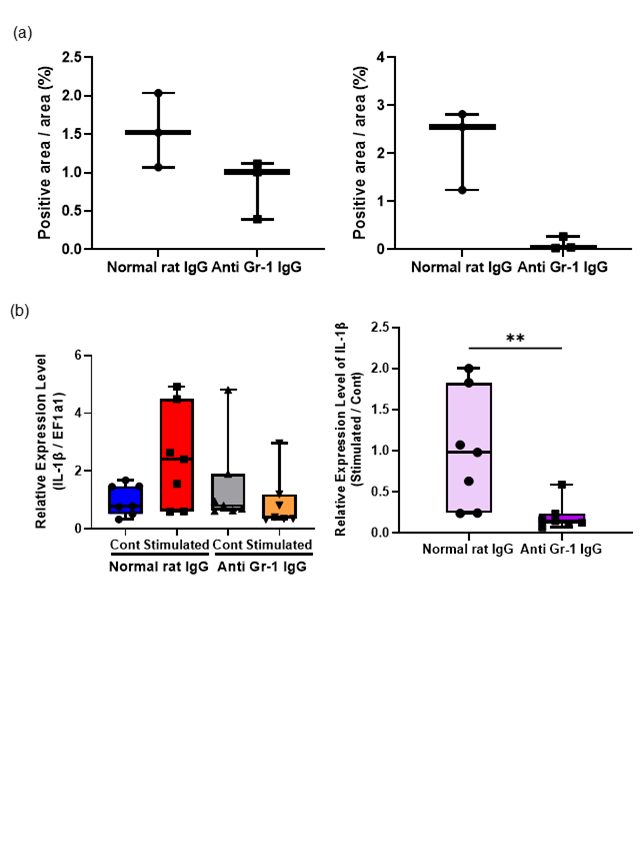


Figure S4. Effects of neutrophil depletion on NETs induction and IL1β expression in the TSM tissues associated with muscle hyperalgesia.

(a) Quantification of citH3 (left) and Gr1-positive areas (right) in the IHC images of the stimulated TSM tissues on day 7 with or without neutrophil depletion (n = 3). (b) qRT-PCR analysis was performed to evaluate expression levels of IL-1β in the TSM tissues on day 7 in sham-stimulated control and stimulated mice with or without neutrophil depletion (n = 7), and was further calculated as a ratio in ipsilateral side relative to contralateral (unstimulated) side of the same individual. All data are shown as Tukey’s boxplots with individual data points. Statistical significance by paired t-test is indicated with ** (p<0.01).


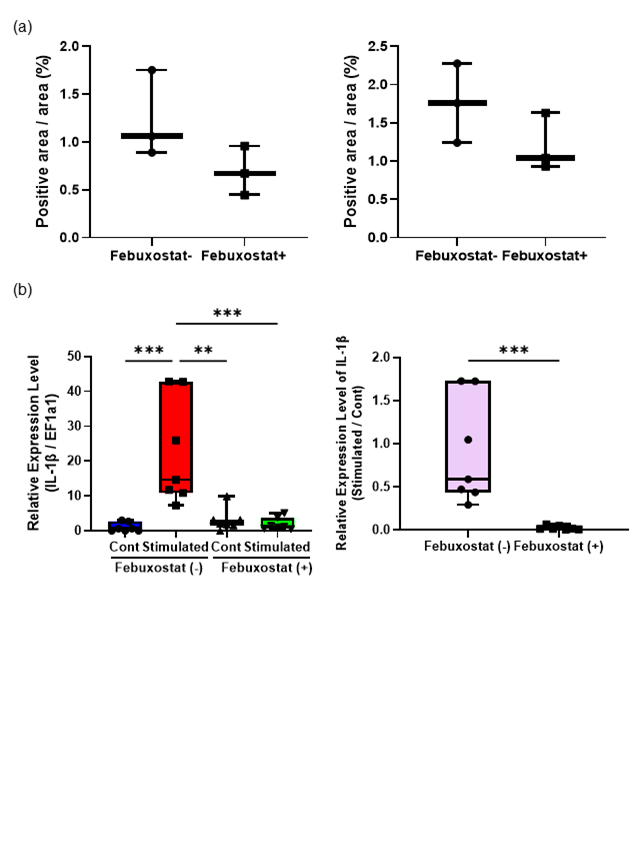
Figure S5

Figure S5. Effects of febuxostat treatment on NETs induction and IL-1β expression in the TSM tissues associated with muscle hyperalgesia.

(a) Quantification of citH3 (left) and Gr1-positive areas (right) in the IHC images of the stimulated TSM tissues on day 7 with the intraperitoneal injection of 5 mg/kg BW/day of febuxostat or a vehicle (n = 3). (b) qRT-PCR analysis was performed to evaluate expression levels of IL-1β in the TSM tissues on day 7 in sham-stimulated control and stimulated mice injected intraperitoneally with 5 mg/kg BW/day of febuxostat or a vehicle (n = 7), and was further calculated as a ratio in ipsilateral side relative to contralateral (unstimulated) side of the same individual. All data are shown as Tukey’s boxplots with individual data points. Statistical significance by paired t-test is indicated with ** (p<0.01) and *** (p<0.001).
