# Supplementary material for: Tissue accumulation of neutrophil extracellular traps mediates muscle hyperalgesia in a mouse model
Source: Sci Rep. 2022 Mar 9;12:4136. doi: 10.1038/s41598-022-07916-8 (PMC8907237; doi:10.1038/s41598-022-07916-8)
Supplement: Supplementary file 4 — Supplementary Video Legend. [file 41598_2022_7916_MOESM4_ESM.docx]

Supplemental Movie 1. Multiphoton microscopy imaging of neutrophil recruitment releasing extracellular DNA due to repeated electrical stimulation (EPS) as assessed by in vivo immunostaining. This movie shows sequential sliced images of the triceps surae muscles tissues, indicating neutrophils (red), extracellular DNA (green) in the TSM tissues (muscle fibers and myofascial collagen fibers, blue) of sham-control (A) or stimulated mice (B) on day 7. Muscle and collagenous fibers are visualized with second-harmonic generation (blue). The yellow structure in the movie is where the red (neutrophils) and green (DNA) coexist, probably indicating NETs. 3D image stacks consisting of 65 optical sections, sized 250×250 μm and scanned at 0.5µm/pixel were acquired with 0.80μm z-steps and reconstructed with Fiji software.
