# Supplementary material for: Tissue accumulation of neutrophil extracellular traps mediates muscle hyperalgesia in a mouse model
Source: Sci Rep. 2022 Mar 9;12:4136. doi: 10.1038/s41598-022-07916-8 (PMC8907237; doi:10.1038/s41598-022-07916-8)
Supplement: Supplementary file 5 — Supplementary Information. [file 41598_2022_7916_MOESM5_ESM.pdf]

Tissue accumulation of neutrophil extracellular traps mediates muscle hyperalgesia in a mouse model

Kazuaki Suzuki[1,2], Masahiro Tsuchiya[3*], Shinichiro Yoshida[1], Kazumi Ogawa[1,2], Weijian Chen[2], Makoto Kanzaki[2], Tadahisa Takahashi[1,2], Ryo Fujita[1,2], Yuqing Li[1,2], Yutaka Yabe[1], Toshimi Aizawa[1], and Yoshihiro Hagiwara[1]

[1] Department of Orthopedic Surgery, Graduate School of Medicine, Tohoku University, Sendai, Japan

[2] Graduate School of Biomedical Engineering, Tohoku University, Sendai, Japan

[3] Department of Nursing, Tohoku Fukushi University, Sendai, Miyagi, Japan

[*]***Corresponding author***

Masahiro Tsuchiya, DDS., PhD.

Department of Nursing, Tohoku Fukushi University

6-149-1 Kunimi-ga-oka, Sendai 981-3201, Japan

Tel: +81-22-728-6049, Fax: +81-22-233-3113

E-mail: tsuchiya-thk@umin.ac.jp

Figure 1

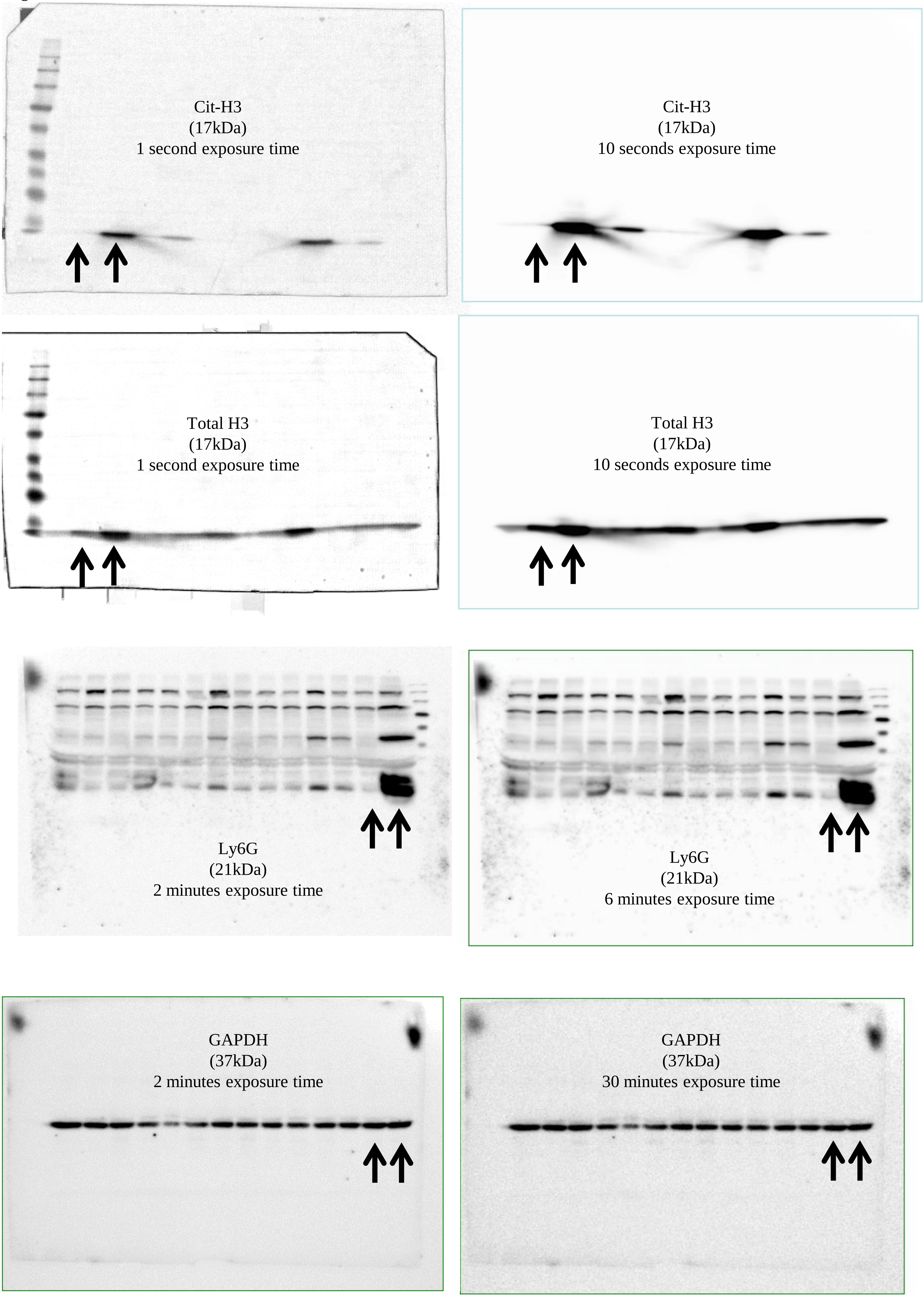

Cit-H3
(17kDa)
1 second exposure time
Cit-H3
(17kDa)
10 seconds exposure time
Total H3
(17kDa)
1 second exposure time
Total H3
(17kDa)
10 seconds exposure time
Ly6G
(21kDa)
2 minutes exposure time
Ly6G
(21kDa)
6 minutes exposure time
GAPDH
(37kDa)
2 minutes exposure time
GAPDH
(37kDa)
30 minutes exposure time

Figure 2

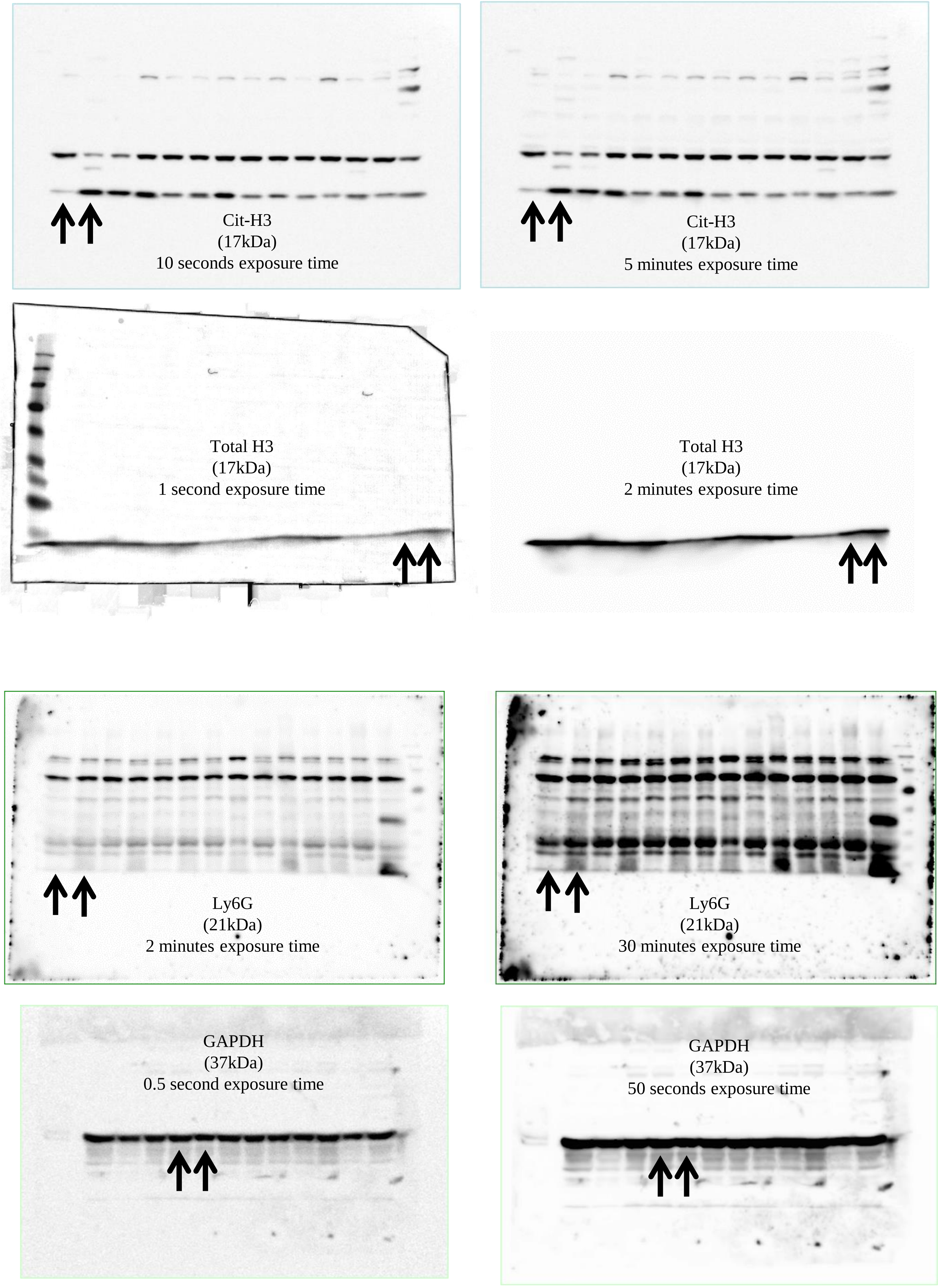
Cit-H3
(17kDa)
10 seconds exposure time
Cit-H3
(17kDa)
5 minutes exposure time
Total H3
(17kDa)
1 second exposure time
Total H3
(17kDa)
2 minutes exposure time
Ly6G
(21kDa)
2 minutes exposure time
Ly6G
(21kDa)
30 minutes exposure time
GAPDH
(37kDa)
0.5 second exposure time
GAPDH
(37kDa)
50 seconds exposure time

Figure 3

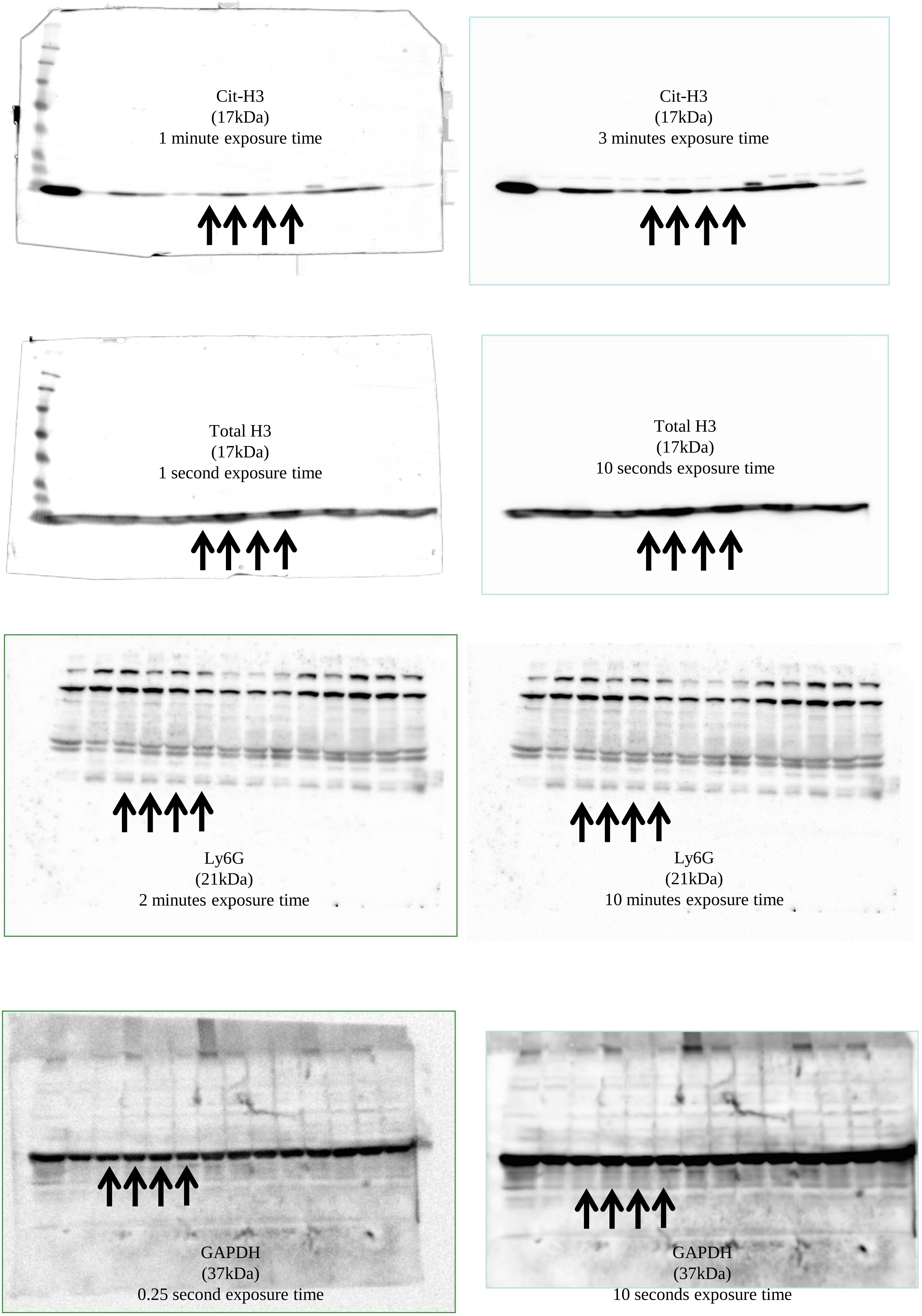
Cit-H3
(17kDa)
1 minute exposure time
Cit-H3
(17kDa)
3 minutes exposure time
Total H3
(17kDa)
1 second exposure time
Total H3
(17kDa)
10 seconds exposure time
Ly6G
(21kDa)
2 minutes exposure time
Ly6G
(21kDa)
10 minutes exposure time
GAPDH
(37kDa)
0.25 second exposure time
GAPDH
(37kDa)
10 seconds exposure time

Figure 4

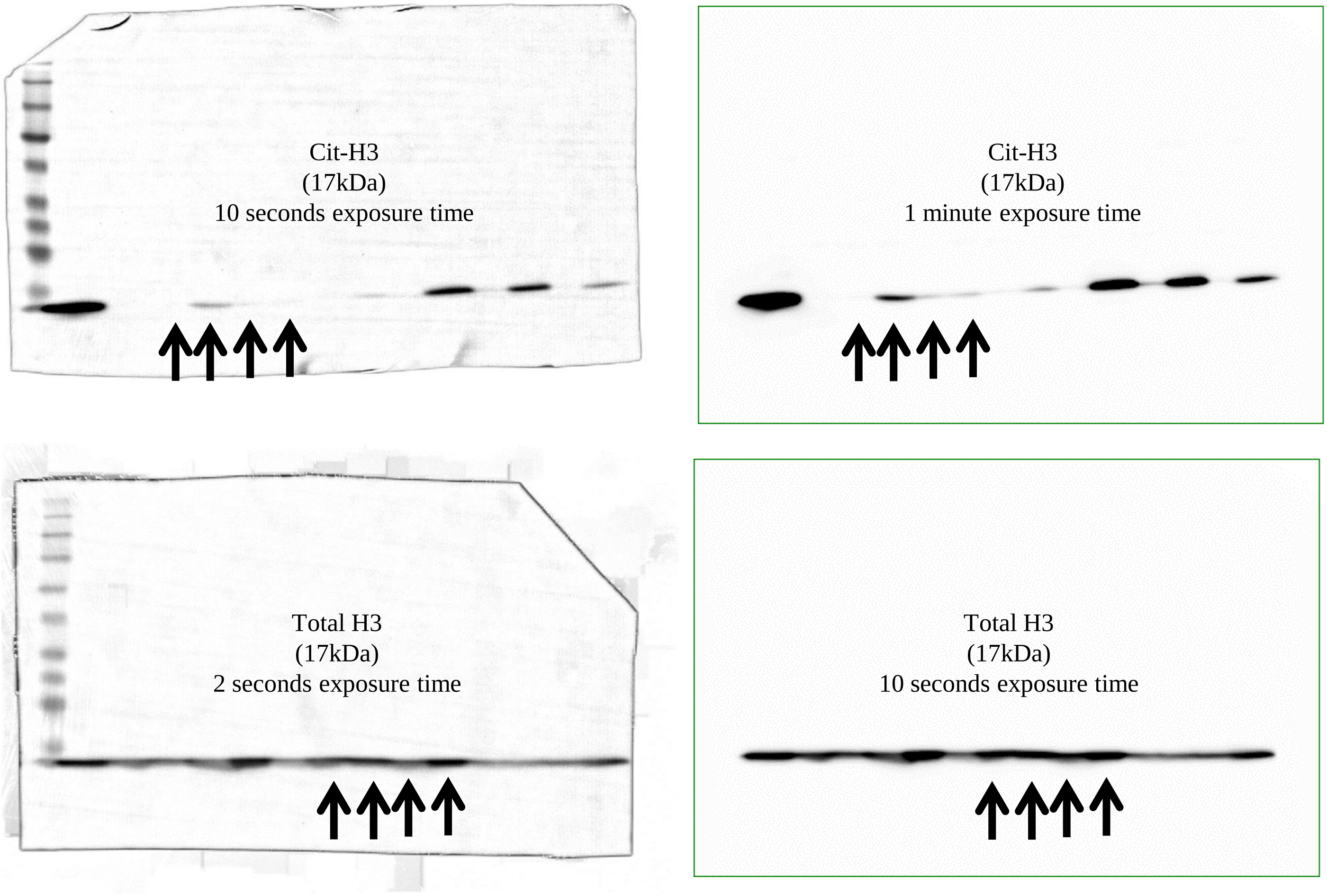
Cit-H3
(17kDa)
10 seconds exposure time
Cit-H3
(17kDa)
1 minute exposure time
Total H3
(17kDa)
2 seconds exposure time
Total H3
(17kDa)
10 seconds exposure time

Figure 5

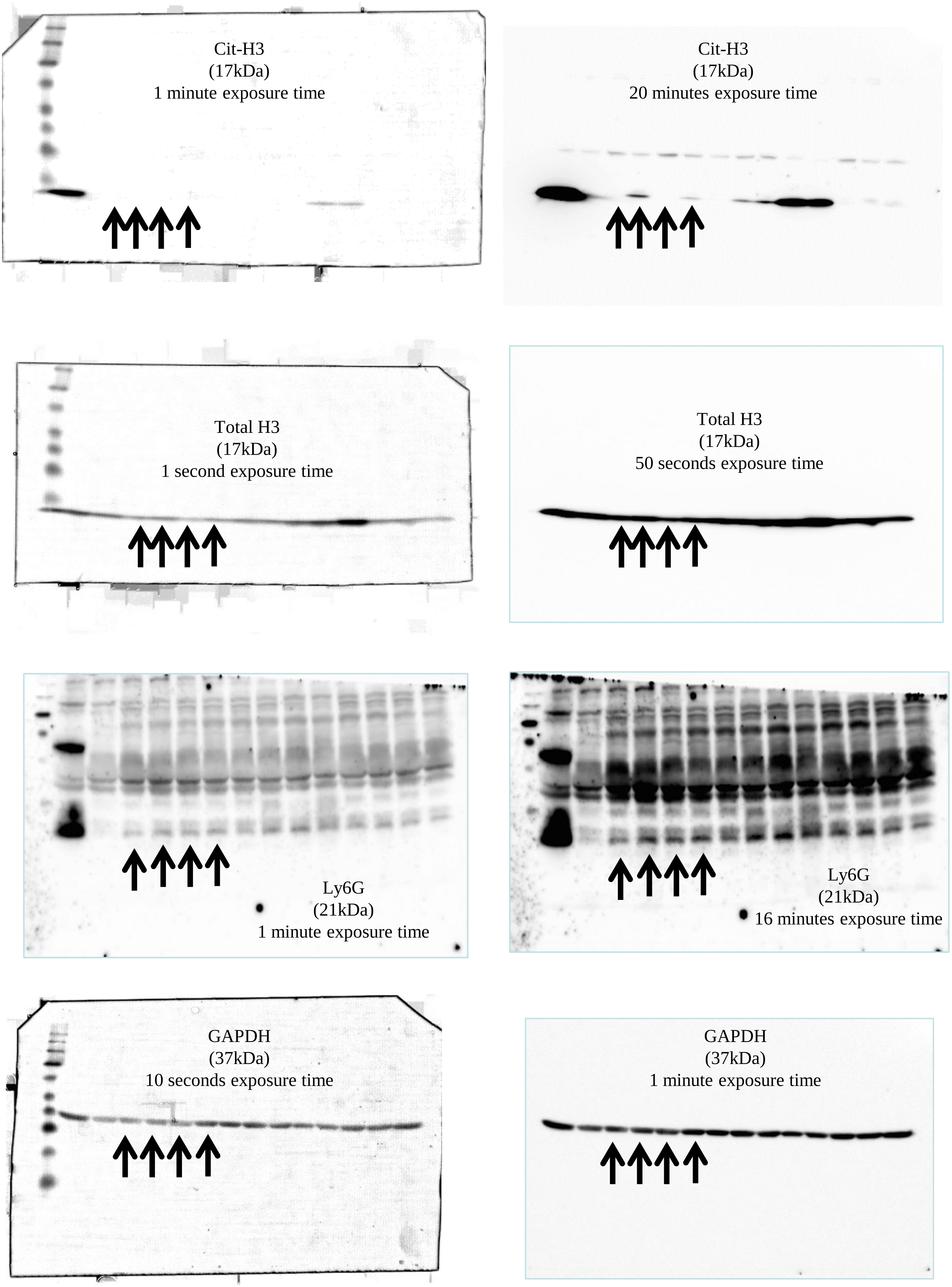
Cit-H3
(17kDa)
1 minute exposure time
Cit-H3
(17kDa)
20 minutes exposure time
Total H3
(17kDa)
1 second exposure time
Total H3
(17kDa)
50 seconds exposure time
Ly6G
(21kDa)
1 minute exposure time
Ly6G
(21kDa)
16 minutes exposure time
GAPDH
(37kDa)
10 seconds exposure time
GAPDH
(37kDa)
1 minute exposure time
